# Harnessing the T Cell to Treat Multiple Myeloma: Dawn of a New Therapeutic Paradigm

**DOI:** 10.3389/fonc.2022.925818

**Published:** 2022-06-24

**Authors:** Alana L. Keller, Daniel W. Sherbenou, Peter A. Forsberg, Tomer M. Mark

**Affiliations:** ^1^ Division of Hematology, Department of Medicine, University of Colorado Anschutz Medical Campus, Aurora, CO, United States; ^2^ Comprehensive Cancer Center, University of Colorado, Aurora, CO, United States

**Keywords:** bispecific antibodies, CAR T cell therapy, T cells, B cell maturation antigen, immunotherapy, myeloma

## Abstract

Multiple myeloma is an incurable hematologic malignancy. The typical disease course for myeloma patients is characterized by initial response to treatment followed by eventual development of resistance. Subsequent cycles of remission and relapse proceed as long as patients have new lines of therapy available to them. This reality has prompted development of many novel immunotherapeutics. Many of these drugs exploit the cytotoxic capabilities of the patients’ own T cells, effectively redirecting them to myeloma cells that are otherwise evading immune attack. Approaches including CAR T cell therapy and bispecific antibodies have displayed impressive efficacy in clinical trials for myeloma patients. This review examines the different approaches that utilize T cells in multiple myeloma therapy and investigates the benefits and risks of these exciting new strategies.

## Introduction

Multiple myeloma (MM), a cancer of plasma cells, is the second most common hematologic malignancy in the United States after non-Hodgkin lymphoma, accounting for approximately 35,000 new diagnoses each year (1). The uncontrolled proliferation of clonal plasma cells in MM leads to anemia, lytic bone lesions that easily fracture, renal failure, impaired humoral immunity, hypercalcemia, and early death (2, 3). MM is a disease of older persons, with a median age at diagnosis of 70. Unfortunately, the disease is incurable in the vast majority of those afflicted leading to over 12,000 deaths yearly in the United States (1). The incidence of MM has been gradually rising in recent decades, with an increase of 126% in cases globally from 1990 to 2016 (4).

The first treatments used for MM, dating from the 1940s, included nitrogen mustard-based alkylating chemotherapies, anthracycline compounds, and glucocorticoids (5, 6). With these agents, life expectancy averaged only 20 months, and extended courses of treatment could not be given due to adverse effects such as chemotherapy-induced myelodysplastic syndrome and opportunistic infections (6). Autologous stem cell transplant (ASCT), using high dose melphalan for myeloablation followed by hematopoietic stem cell rescue, became a widespread treatment modality in the 1990s as it was the first treatment shown to have a survival benefit in MM in randomized clinical trials and confirmed by meta-analysis (7–10). However, the survival benefit of ASCT is modest, averaging 11 months, and adverse events such as prolonged fatigue, immunosuppression, and delayed blood count recovery limited its use to those patients who were generally fitter and younger.

The late 1990s and early 2000s saw the advent of the “novel agents” to treat MM: proteasome inhibitors (PIs) and immunomodulatory agents (IMiDs) (11). Bortezomib was the first PI to be used in the clinic leading to responses and improved overall survival in the treatment of both upfront and relapsed MM. Other PIs, carfilzomib and ixazomib, with different chemical structures and binding properties to the proteasome, have since been FDA approved and incorporated into clinical use (12). Thalidomide was the first IMiD to be used clinically for MM, initially tried at the urging of a patient’s wife after she had read that thalidomide had anti-angiogenic properties (13). A derivative of thalidomide, lenalidomide, was later shown to be both directly cytotoxic to the MM cells and also a powerful activator of T cells, potentially leading to more MM cell death (14, 15). The observation that use of lenalidomide maintenance therapy after allogeneic stem cell transplant leads to increased incidence and severity of graft vs host disease points to the T cell activating properties of IMiDs (16). In addition to T cell activation, lenalidomide also alters the cytokine milieu to decrease the inflammation which fuels MM growth through inhibition of IL-6 and TNF-alpha secretion (17). A later derivative of thalidomide, named pomalidomide, was demonstrated to have efficacy even in the setting of lenalidomide resistance (18). Unfortunately, while overall survival for MM has certainly improved with use of these novel agents, it has historically been dismal once patients become refractory to both IMiD and PI therapy with a median overall survival of only 13 months (19).

Monoclonal antibodies were the first immunotherapeutics in MM, the application of which we have reviewed previously (20). These have arrived relatively recently, with the first monoclonal antibody (MoAb) against CD38, daratumumab, gaining FDA approval in the use of multiply-relapsed or refractory multiple myeloma (RRMM) in 2015 based on a single-agent response rate of 30% (21, 22). CD38 is a cell surface protein present on plasma cells and red blood cells that acts both as an adhesion molecule and an ectoenzyme involved in calcium metabolism (23, 24). In RRMM, daratumumab added in combination with either IMiDs led to improved response rates and progression free survival over the backbone regimens alone in multiple studies (25–27). In subsequent studies, when daratumumab is combined with either IMiDs or PIs (or both) as part of initial treatment for newly diagnosed MM, the overall response to treatment rises dramatically with nearly 90% of patients achieving tumor reduction and 20-30% reaching minimal residual disease negativity by next-generation sequencing or flow cytometry-based detection (28–33). After daratumumab, isatuximab became the second anti-CD48 MoAb FDA-approved for use in MM (34). Isatuximab is an anti-CD38 monoclonal antibody approved for relapsed or refractory MM in combination with either pomalidomide or carfilzomib (35, 36). Moving beyond targeting CD38, elotuzumab is another monoclonal antibody approved for MM instead directed against Signaling Lymphocytic Activation Molecule Family member 7 (SLAMF7) (37, 38). The clinical observation that elotuzumab does not work well as a single agent, but can reduce chance of relapse by 30-50% in combination with lenalidomide or pomalidomide, supports the important role of NK and T cell activation in anti-tumor efficacy (39–41). All in all, success of daratumumab, isatuximab, and elotuzumab served as proof-of-concept for the many adaptive immune-mediated therapies being developed for relapsed/refractory myeloma.

Following the discovery of the power of the immune checkpoint inhibitors in other malignancies, and the success of T-cell activating treatments in other hematologic malignancies, there was an intensive effort to exploit T cells in combating myeloma. What follows is a discussion of the evolution of immunotherapy in MM with a focus on T cell-directed strategies that have been tested in MM, including immune checkpoint inhibitors, bispecific antibodies, and chimeric antigen receptor (CAR) T cells.

## Immune Checkpoint Inhibitors

Cytotoxic CD8^+^ T cells require two signals to become activated to kill a foreign, infected, or cancerous cell. The first signal is engagement of the T cell receptor (TCR) with major histocompatibility complex (MHC) Class 1 expressed on the target cell (42). A second signal results from CD28 engagement on the T cell with CD80/86 on professional antigen-presenting cells (APCs), which is required to promote ongoing T cell stimulation and survival (42). This requirement for T cell co-stimulation has been exploited in anti-cancer therapy in two different ways. The first is *via* the inhibition of cytotoxic T-lymphocyte-associated protein 4 (CTLA4), which is found on CD4 and CD8 T cells (43). The expression of CTLA4 increases with the level of TCR activation and general T cell stimulation *via* cytokines such as IL-2 (44). CTLA4 has a higher affinity for co-stimulatory ligands CD80/86 on APCs than CD28, thus outcompeting and acting as a brake on T cell activation (45). It follows that blockade of CTLA4 on T cells would lead to increased activity of cytotoxic T cells and more tumor killing. This has turned out to be the case for certain tumor types and the anti-CTLA4 antibody, ipilimumab, has been FDA approved for the treatment of metastatic melanoma (46).

Another means by which cancer cells evade T cell immune surveillance is *via* binding to the programmed cell death 1 (PD-1) protein. PD-1 is a cell surface protein expressed on T-lymphocytes, acting as another brake on their activation *via* interaction with its ligand PD-L1. This interaction can lead to T cell exhaustion and differentiation to regulatory T cells. Physiologically, PD-L1 is expressed on most normal cells, and abnormal expression may be linked to autoimmune disease *via* unchecked T cell activity (47). Pathologically, PD-L1 can be aberrantly expressed by tumor cells, including MM cells, to avoid this normal checkpoint that identifies and eliminates abnormal, cancerous cells. Monoclonal antibodies against PD-L1 and PD-1 have been developed, such as pembrolizumab, nivolumab, atezolizumab, and durvalumab, and are in clinical use either alone or in combination with anti-CTLA4 treatment or chemotherapy for several malignancies, including bladder cancer, renal cell carcinoma, and lung cancer. A predictable adverse effect of checkpoint blockade would be development of auto-reactive T cells. Indeed, this has been observed in clinical practice, with treatment-related side effects including thyroiditis, pneumonitis, and in rare cases cerebritis. Interestingly, it appears that patients with immune-related adverse events during PD-1 axis blockade treatment may have better tumor responses (48).

CTLA4 and PD-L1 have both been found to be highly expressed in bone marrow samples from MM patients, however the focus thus far on checkpoint inhibition treatment for MM has been on the PD-1 pathway (49, 50). A phase 1b study of nivolumab (anti-PD-1 MoAb) was performed in advanced hematologic malignancies, with a subset of 27 participants with relapsed MM (51). Of these patients, 60% had stabilization of the MM for 11 weeks, but none had significant reduction in tumor burden. Postulating that more T cell stimulation would be useful to enhance tumor response, PD-1 inhibition was combined with IMiD treatment. In the initial phase 1 study [KEYNOTE-023] of pembrolizumab (another anti-PD-1 MoAb) and lenalidomide for relapsed MM, there was an overall response rate of 44% with an additional 50% of patients achieving stable disease; the 1-year survival rate was 82.6% (52). Notably, 93% of the patients involved in this study had been exposed to and progressed after prior lenalidomide treatment. Another phase 1/2 study of pembrolizumab, pomalidomide, and dexamethasone in more heavily pre-treated MM [HD-00061522] produced an impressive response rate of 60% and median PFS of 17.4 months, albeit most patients in this study had not been previously treated with pomalidomide (53). Although very promising initially, randomized studies of pembrolizumab with or without IMiDs were halted by the FDA in 2019 due to excess deaths in those who received the checkpoint inhibitor. In the phase 3 study of lenalidomide/dexamethasone with or without pembrolizumab as initial treatment in newly diagnosed MM, [KEYNOTE-185], overall responses between the control and pembrolizumab arms were similar at 62% vs 64%, and 82% vs 87% of subjects were without disease progression at 6 months respectively (54). Nine out of 51 (19%) patients in the pembrolizumab arm had died during the study, as compared to only 6% in the control arm. In the relapsed setting, the phase 3 study of pomalidomide/dexamethasone with or without pembrolizumab [KEYNOTE-183] showed an inferior overall response rate in the investigational arm (34% vs 40% for pomalidomide/dexamethasone alone), and again there were more deaths in the pembrolizumab arm at 23% vs 17% (55). Notably, approximately a third of the deaths in the pembrolizumab arms of both KEYNOTE phase 3 studies were due to immune-related adverse events such as myocarditis or Stevens-Johnson syndrome. Increased infection rates were also noted. Thus, in MM compared to other tumors, it appears that the risks of checkpoint inhibition outweigh the benefits, at least when combined with IMiDs. Further development of immune checkpoint strategies in MM treatment was slowed significantly and a role for checkpoint inhibitors in MM at this point appears unlikely.

## Bispecific Antibodies

Bispecific antibodies (bsAbs) are engineered, bivalent monoclonal antibodies that comprise a diverse ‘zoo’ of options displaying great variation in structure (56). The earliest formats included Bispecific T cell engagers (BiTEs), which are comprised of two single-chain variable fragments (scFv) connected by a short, flexible linker. Blinatumumab, a CD19-targeting BiTE used in acute lymphoblastic leukemia, was the first bsAb approved in oncology (57, 58). However, these constructs have a short half-life and require burdensome continuous intravenous infusions (59). More recently, the prominent agents in trials take forms more similar to MoAbs, in which Fc regions are included to prolong molecule half-life and allow for periodic dosing (60, 61). Generally, bsAbs can be separated into groups based on their binding partners: (1) those that bind two immune targets, (2) those binding two tumor-associated antigens (TAA), and (3) those that bind one TAA and one immune target (62, 63). The majority of bsAbs in development for MM belongs to the third category, with the anti-MM cell targeting arm binding the TAAs including BCMA, FcRH5, or GPRC5D (64). As shown in **Table 1**, we will highlight the bsAbs furthest in clinical development for each target.

**Table 1:** Preliminary Clinical Data of Bispecific Antibodies in Multiple Myeloma.

Drug	Targets	Company	Grade 3-4 TEAE	CRS	Response Rate	Survival (e.g. PFS, DOR, OS)
Elranatamab (PF-06863135) (65)	anti-CD3/BCMA	Pfizer	neutropenia 60%, anemia 38%, lymphopenia 64%, thrombocytopenia 31%	83%	At RP2D- ORR: 83%; sCR: 83%	median DOR not reached
REGN5458 (66)	anti-CD3/BCMA	Regeneron	anemia 9%, lymphopenia 7%, infections 20%	38%	At all doses-ORR: 36%; CR: 31%	43.8% of responders DOR > 4 m; 18.8% responders DOR > 8 m
Teclistamab (67)	anti-CD3/BCMA	Janssen	neutropenia 45%, anemia 27%, thrombocytopenia 18%, fatigue 2%	At RP2D- 67%	At RP2D- ORR: 65%; ≥VGPR: 60%; ≥CR: 40%	median DOR not reached; 6 m DOR: 90% (95% CI 63-97)
AMG 701 (68)	anti-CD3/BCMA	Amgen	CRS 7%, atrial fibrillation 1%, acidosis 1%, thrombocytopenia 1%	61%	At 3-12mg- ORR: 36%; sCR: 4%	median DOR: 3.8 m (ongoing in 14/17 pts); maximum DOR: 23 m
TNB-383B (69)	anti-CD3/BCMA	TeneBio/ Abbvie	CRS 3%	52%	At ≥40 mg- ORR: 64%; ≥VGPR: 43%; CR: 16%	At ≥40 mg- ORR: 64%; ≥VGPR: 43%; CR: 16%
AMG420 (70)	anti-CD3/BCMA	Amgen	CRS 2%, polyneuropathy 5%, edema 2%	38%	At all doses- ORR: 31%; ≥CR: 21%	median DOR > 8.4 m
CC-93269 (71)	anti-CD3/BCMA	Celgene	neutropenia 43%, anemia 37%, infections 30%, thrombocytopenia 17%	77%	At all doses- ORR: 43%; ≥CR: 17%	DOR: 5.3-40.6 m
Talquetamab (72)	anti-CD3/GPRC5D	Janssen	405 µg/kg weekly: CRS 3%, neutropenia 60%, infections 3% 800ug/kg biweekly: neutropenia 35%, infections 4%	405 µg/kg weekly: 73% 800ug/kg biweekly: 78%	At 405 µg/kg weekly- ORR: 70%; ≥VGPR: 57% At 800 µg/kg biweekly dose- ORR: 71%; ≥VGPR rate: 53%	6 m DOR for pts given 405ug/kg dose: 67% [95% CI: 41-84]; median DOR not reached
Cevostamab (BFCR4350A) (73)	anti-CD3/FcRH5	Genentech	CRS 1%, anemia 22%, neutropenia 16%, infections 19%	80%	At 160mg dose- ORR: 55% At 90mg dose- ORR: 37%	median DOR: estimated 15.6 m (95% CI: 6.4-21.6 m)

TEAE, treatment-related adverse events; CRS, cytokine release syndrome; RP2D, recommended phase 2 dose; ORR, overall response rate; sCR, stringent complete response; CR, complete response; VGPR, very good partial response; PFS, progression-free survival; DOR, duration of response; OS, overall survival; m, month; yr, year; pts, patients.

There are currently several BCMA-directed bsAbs showing promise in ongoing clinical trials. The first bispecific antibody tested clinically in MM was AMG-420, a BiTE directed against CD3/BCMA that showed an excellent overall response rate of 70% in a phase I trial for relapsed or refractory MM (70). However, 2 weeks of continuous intravenously infused AMG-420 was onerous and further development was abandoned. Instead, AMG-420 was reformulated to AMG-701, a bsAb with an Fc region and once-weekly dosing that produced an impressive overall response of 83% at target dosing (68). However, since AMG-420 results became public, the competition has intensified in anti-MM bsAbs. In a phase 1/2 clinical trial of teclistamab (anti-CD3/BCMA), patients who had progressive disease were treated after a median of 5 prior lines of therapy, and an overall response rate of 65% was seen at the recommended phase 2 dosing (67). While overall follow-up has been short, it is encouraging that among the responders, over 90% maintained the response for 6.5 months. These results appear to be corroborated in the phase 2 extension. Another anti-CD3/BCMA bispecific, elranatamab, displayed high response rates in a phase 1 clinical trial in patients with a median of 6 prior lines of therapy (65). The observed overall response rate of patients receiving the recommended phase 2 dose (RP2D) was 83%. Remarkably, 75% of patients previously treated with BCMA-targeted therapies still achieved response. Other anti-BCMA bsAbs such as REGN5458, TNB-383B, and CC-93269 have demonstrated initial promise as well (**Table 1**). Given the diversity of anti-BCMA bsAbs in clinical development, it is unclear how many will proceed through late-phase investigation and how differences in described efficacy, dosing schedule, and toxicity profiles may ultimately drive clinical utilization.

In addition to BCMA, other MM antigens have emerged as promising targets for bsAbs. Fc Receptor Homolog 5 (FcRH5) is another attractive target, as it is expressed exclusively in B-lineage cells, mature plasma cells, and MM cells (74). FcRH5 is a protein that plays a role in isotype selection and proliferation in activated B cells (75). In a phase 1 study of cevostamab (anti-CD3/FcRH5) in patients with a median of 6 prior lines of therapy, 55% achieved response at the higher dose level of 160mg, and estimated median duration of response was 15.6 months (73). Another target, the orphan G protein coupled-receptor class C group 5 member D (GPRC5D), is a cell surface protein that is highly expressed on malignant plasma cells as well as in hard keratinized tissues such as hair and nails. The function of GPRC5D is currently unknown, although its high expression correlates with poor prognosis in MM patients (76). Updated results of a phase 1 study of talquetamab (anti-CD3/GPRC5D) in subjects with relapsed/refractory multiple myeloma (RRMM) were excellent, with a response rate of 70% seen in the 405 µg/kg weekly RP2D cohort and 71% at the 800 µg/kg every-other-week RP2D (72). It is also worth noting that due to off-target GPRC5D expression on keratinized tissues, oral and dermatologic adverse events were frequently observed, but they have been described as manageable (77). Initial testing has begun to explore the safety and efficacy of various MM bsAbs in combination with additional agents including MoAbs and other bsAbs with different TAAs. In summary, there are several bispecific antibodies showing very promising results in early clinical trials, and data from larger randomized studies is eagerly anticipated.

## CAR T Cells

Historically, cell-based therapies in MM have consisted of stem cell transplants. High-dose myeloablative chemotherapy with autologous stem cell transplantation (ASCT), has been used since the early 1990s as a means to achieve tumor reduction. ASCT has been a long-standing standard of care as a result of randomized clinical trials showing a survival benefit compared to conventional chemotherapy (7, 8, 78). Despite often deep and durable responses after ASCT, relapse is largely inevitable, with a median response of 54 months when administered with high dose chemotherapy and 50 months with supporting lenalidomide, bortezomib, and dexamethasone (8, 78). The use of post-transplant lenalidomide maintenance extended response to a median of 40 months (79, 80). Allogeneic stem cell transplant has also been tested in MM to induce a graft vs tumor effect mediated by donor T cells. However, there is a high treatment-related mortality in up to 40% of patients in early studies. With more current approaches, estimates of treatment-related mortality are between 5-10% due to complications of acute graft vs host disease (GvHD) and prolonged immunosuppression (81, 82). Furthermore, although graft vs tumor effect has been demonstrated in MM by response to donor lymphocyte infusion or lifting of immunosuppression after allogeneic stem cell transplant, graft vs tumor effect is relatively weaker in MM when compared to other hematologic malignancies (83, 84). Until recently, the development of novel cell-based therapies was limited.

Chimeric antigen receptor (CAR) T cells are a new cellular therapy in MM that utilize autologous engineered T cells for anti-tumor effect. CAR T cells are T lymphocytes with an artificial receptor engineered to target a specific TAA. A CAR construct allows a patient’s T cells to attack their own malignant cells in an MHC-independent fashion, bypassing the tumor’s immune evasion mechanisms and avoiding acute and chronic GvHD. CAR T constructs have a single chain variable scFv linked to the TCR transmembrane region and intracellular signal activating domains. The intracellular domain can activate downstream signaling in T cells to promote activation and pro-inflammatory cytokine release (e.g. IL-2, TNF-alpha, IL-6). The intracellular domain of early CAR T constructs was solely CD3ζ, which led to some activity, but limited duration due to lack of a proliferation signal (85). Newer CAR T constructs include additional co-stimulatory domains (most often 4-1BB and CD28) in addition to CD3ζ, greatly enhancing persistence and activation (86, 87). In the process of CAR T cell development, a patient stops any chemotherapies and corticosteroids for a short period, then undergoes lymphocyte apheresis (88, 89). After lymphocyte collection, the cells are sent for CAR T manufacturing *via* lentiviral transduction of a DNA cassette encoding for the chimeric TCR. Once created, CAR T cells are selected and expanded in culture to provide the significant cell number needed for infusion. This process takes approximately 4-6 weeks, during which the patient may receive a therapy “bridge” while waiting for the CAR T product. Once ready, the patient is then given a 3-day course of lymphodepletion chemotherapy, usually fludarabine and cyclophosphamide, to ensure that the infused CAR T cells are not immediately destroyed by the recipient’s immune system (90). The patient is then monitored closely for signs and symptoms of cytokine release syndrome (CRS) and neurotoxicity. These T cell therapy-specific complications are described in a separate section to follow.

CAR T cells have been shown to have remarkable activity in several hematologic malignancies (91). There are four anti-CD19 CAR T products currently approved for use in B cell acute lymphoblastic leukemia (brexucabtagene autoleucel and tisagenelcleucel) and non-Hodgkin B cell lymphomas (axicabtagene ciloleucel and lisocabtagene maraleucel) (92). The first CAR T product for relapsed MM, idecabtagene vicleucel (ide-cel), was approved in 2021 for patients who have received 4 or more prior lines including an IMiD, a PI, and a CD38-directed MoAb. As most MM cells lack CD19, ide-cel is a BCMA-directed CAR T that possesses a 4-1BB costimulatory domain. A phase 1 study of ide-cel showed dose-dependent efficacy in RRMM, with depth and duration of response improving as infused cell numbers increase (93). In 128 patients with MM that progressed after prior PI, IMiD, and anti-CD38 MoAb treatment, ide-cel led to an overall response rate of 73%, with 33% achieving complete remission at doses ranging from 150-450 x 10^6^ CAR T cells/kg. At target dosing of 450x10^6^ CAR T cells/kg, ide-cel had an 83% overall response rate and progression-free survival of 12.1 months. Phase 3 studies comparing ide-cel vs. other standard-of-care treatments for RRMM are currently underway. In early 2022, another BCMA-directed CAR T product with a 4-1BB costimulatory domain, ciltacabtagene autoleucel (cilta-cel), became the second CAR T product approved for use in RRMM with a similar indication as ide-cel. Cilta-cel may have even better activity than ide-cel, potentially due to its bivalent binding region. In a phase 1/2 study of cilta-cel, the overall response to the target dosing of 0.75x10^6^ CAR T cells/kg was 98% in MM patients with a median of six prior lines of therapy (94). The 12-month progression-free survival was excellent at 77% with a 1-year survival rate of 89%. Cilta-cel is also currently being explored in more clinical settings in several ongoing trials.

Beyond ide-cel and cilta-cel, there are several BCMA CAR T cell therapies in clinical trials that use novel manufacturing approaches designed to reduce toxicity and improve response (**Table 2**). P-BCMA-101 is manufactured using a transposon-based technology called piggyBac that favors production of T cells with the stem cell memory phenotype and reduces toxicity (100). The overall response rate of patients in the corresponding phase 1/2 clinical trial was 57% (95). An updated version of the ide-cel product, bb21217, has also been evaluated in early phase trials. Unlike ide-cel, bb21217 is cocultured with a PI3K inhibitor to increase the number of memory-like T cells and remove senescent cells from the CAR T product (96). So far, an ORR of 69% has been observed in a phase 1 trial. Recently the development of both P-BCMA-101 and bb21217 have been discontinued demonstrating some of the challenges of iterative CAR T development. CT053 and CT103A both use a human anti-BCMA scFv and clinical trial results reported ORRs of 87.5% and 100% respectively (97, 98). However, the small patient cohort included in the CT103A phase 1 trial makes it difficult to interpret the high ORR. ALLO-715 is an allogenic CAR T product that is engineered with a modified T cell receptor and CD52 to reduce GvHD in the ‘off-the-shelf’ product (99). Current phase 1 data reflects an ORR of 62%. CAR T cells directed to other MM targets, such as CD38, SLAMF7, GPCR5D, CD56, and CD138 have also been developed, but clinical data has yet to emerge (101). Thus far, although there are clear potential improvements to make upon the lead CAR T products in MM (especially allogenic CAR Ts), these have yet to bear out in trials.

**Table 2:** Clinical Response of BCMA CAR T Cell Therapies in Multiple Myeloma.

Drug	Company	Grade 3-4 TEAE	CRS	Response Rate	Survival (e.g. PFS, DOR, OS)
Idecabtagene vicleucel (ide-cel) (93)	Bristol Myers Squibb/bluebird bio	CRS 5%, Neurotoxicity 3%,	84%	ORR: 73%; CR: 33%	median DOR: 10.7 m; median PFS: 8.8 m (95% CI, 5.6-11.6); OS 78% at 12 m (estimates)
Ciltacabtagene autoleucel (cilta-cel) (94)	Janssen	neutropenia (94.8%), anemia (68.0%), leukopenia (60.8%), thrombocytopenia (59.8%), and lymphopenia (49.5%)	95%	ORR: 98%; ≥VGPR: 95%; sCR: 80.4%	median DOR: 21.8 m – NE; 2-yr PFS: 60.5% (95% CI, 22.8 m – NE)
P-BCMA-101 (95)	Poseida Therapeutics	neutropenia 79%, thrombocytopenia 30%, anemia 30%	17%	ORR: 57%	Responses ongoing
bb21217 (96)	Bristol Myers Squibb/bluebird bio	CRS 1%, neurotoxicity 4%	75%	ORR: 69%; ≥VGPR: 58%; sCR/CR: 28%	estimated median DOR: 27.2 m
CT053 (97)	CARsgen Therapeutics	neutropenia 100%, leukopenia 100%, thrombocytopenia 36%	86%	ORR: 87.5%; CR: 79%	median DOR: 21.8 m
CT103A (98)	Nanjing IASO Biotherapeutics	leukopenia 100%, neutropenia 100%, lymphopenia 100%, anemia 89%, thrombocytopenia 94%	94%	ORR: 100%; CR/sCR: 72%	1-yr PFS: 58.3%
ALLO-715 (99)	Allogene Therapeutics	CRS 2%, infections 13%	52%	ORR: 62%; VGPR: 39%	median DOR: 8.3 m (95% CI: 1.5 – not reached)

TEAE, treatment-related adverse events; CRS, cytokine release syndrome; ORR, overall response rate; CR, complete response; sCR, stringent complete response; VGPR, very good partial response; PFS, progression-free survival; DOR, duration of response; OS, overall survival; m, month; yr, year; NE, not estimable.

While CAR T cell therapy has now been approved in MM, development is ongoing and other modalities are still being explored. For example, TCR-engineered cells are patient-derived T cells modified to target a specific tumor-associated antigen or neoantigen (102). Unlike CARs, whose artificial receptors allow efficacy independent of MHC presentation, which is often downregulated by immunosuppressive tumor cells, TCR-engineered T cells rely on native TCR biology (103). Production of TCR-engineered T cells is a similar process to CAR T production, expensive, and can take approximately 4-6 weeks to produce (102, 104). TCR-engineered T cells have shown early promise in leukemia and are being evaluated in a few MM clinical trials in a small number of patients (102, 105–107). One study, in which TCRs are engineered to target a shared sequence between antigens New York esophageal squamous cell carcinoma-1 (NY-ESO-1) and L-antigen family member 1 (LAGE-1), has reported an objective response rate of 80% at day 42 and median progression free survival of 13.5 months in 25 relapsed/refractory myeloma patients with at least one adverse cytogenetic abnormality (107). This area is rapidly evolving, and more clinical trial data is expected to emerge.

## Toxicities of T Cell Directed Therapy

As discussed in the immune checkpoint inhibition section above, immune-mediated side effects can be significant in patients with MM, likely responsible for the early termination of clinical studies of PD-1 inhibitors. Representing the consequences of T cell hyperactivation, cytokine release syndrome (CRS) and a spectrum of neurologic symptoms dubbed immune effector cell-associated neurotoxicity syndrome (ICANS) are the unique adverse effects of CAR T treatment. Hematologic toxicities (cytopenia, hypogammaglobulinemia) although not unique to T cell modalities, represent other CAR T-associated toxicities resultant from lymphodepletion regimens preceding CAR T infusion (108). CRS has been seen with CAR T, bispecific antibodies, and haploidentical (5/10 HLA matched) allogeneic stem cell transplant. It can also occur as an adverse effect from checkpoint inhibitors and anti-thymocyte globulin (ATG), albeit much less frequently. The symptoms of CRS unfold when unchecked T cell activity leads to an outpouring of pro-inflammatory cytokines into the circulation (IFN-gamma initially, then IL-6, TNF-alpha, and IL-10), resulting in a sepsis-like syndrome with fevers and potential for distributive shock characterized by hypotension, delirium, disseminated intravascular coagulation, hypoxia, and even death without treatment (48). The role of IL-6 in CRS is paramount, as demonstrated by the success of the neutralizing treatment tocilizumab, a MoAb which blocks the IL-6 receptor.

The earlier tocilizumab is given the in course of CRS, the less severe and durable the CRS. Fortunately, it does not appear that clinical efficacy of the CAR T infusion is hindered by early tocilizumab (109). The vast majority of CRS is mild and self-limited, but the timing of onset and duration can vary depending on the therapy used. In the pivotal KarMMa-2 trial ide-cel was associated with an 84% rate of any grade CRS and only 5% rate of grade 3 or higher CRS (93). The median time to onset of CRS was 1 day with a median duration of 3 days. In the CARTITUDE-1 trial cilta-cel was associated with a 95% rate of any grade CRS and only 4% rate of grade 3 or higher CRS (110). Median time to onset of CRS was 7 days and median duration was 4 days. For bsAbs, AMG701 had an overall rate of CRS of 61%, only 7% of moderate severity or greater; talquetamab was associated with a 73% rate of CRS at the 405 µg/kg weekly dose and 78% at the 800µg/kg biweekly dose with very few cases that were more than mild in intensity (68, 72). The anti-FcRH5 bispecific cevostamab had a slightly higher reported rate of CRS at 80%, but also with few severe cases (73). For the bsAbs, the onset of CRS is quick with intravenous vs subcutaneous administration (24 vs 48 hours) and median duration of symptoms likewise was 24-48 hours (65, 68, 111, 112). Due to similar findings, many clinical trials for bsAbs in MM have attempted different steps including planned dose escalation steps and use of subcutaneous administration in attempts to reduce CRS rate and severity. In most cases, CRS tended not to recur with dosing beyond the first infusion at target dose. Unfortunately, the unpredictable and wide range of timing for CRS complicates these T-cell activating treatments. Currently, many patients undergoing CAR T are hospitalized for a planned observation period, although there is effort being made now to dose and manage CAR T in the outpatient setting.

ICANS, another commonly observed toxicity, may present as headache, confusion, difficulty with word finding and speech, and in severe cases seizures, encephalopathy and obtundation. ICANS may be difficult to parse from CRS and both issues may occur simultaneously. The pathophysiology of ICANS is still unclear, but evidence from a mouse model suggested it may occur through inflammatory changes to the endothelium at the blood-brain barrier, leading to capillary leak of inflammatory cytokines and clotting factors into the central nervous system (113). For bsAbs teclistamab and talquetamab the ICANS rate was low, at 3% and 6%, respectively (67, 112). Cevostamab, on the other hand, was associated with a higher rate of neurotoxicity at 13% (73). In the case of CAR T treatment, it appears that there may be an association between the use of a CD28 costimulatory domain and risk of ICANS, as these CAR T products in use for treatment of lymphoma generally have a higher rate and severity of ICANS than the 4-1BB CAR T cells. The CD28 CAR T products axicabtagene ciloleucel and brexucabtagene autoleucel (used for non-Hodgkin lymphomas) had neurotoxicity rates of approximately 65%, compared to approximately 20% for the 4-1BB CAR T products idecabtagene and ciltacabtagene (93, 110, 114, 115). In KarMMa-2 ide-cel had an associated rate of 18% with only 3% of patients experiencing grade 3 events, median time to onset of events was 2 days with a median duration of 3 days (93). Cilta-cel has been associated with a neurotoxicity rate of 21% with 9% grade 3/4 events (110). In CARTITUDE-1 the neurotoxicity observed with Cilta-cel included both ICANS in 17% of patients with median time to onset of 8 days and median duration of 4 days as well as other neurotoxicity in 12% of patients, all of whom had previous CRS and 2/3s of whom had prior ICANS. This other neurotoxicity occurred later with a median onset of 27 days with variable associate symptoms including a cluster of movement and neurocognitive treatment-emergent events. Further exploration to understand and minimize the frequency to this other neurotoxicity is ongoing and a recent description of BCMA expression on neurons and astrocytes in the basal ganglia may represent a possible mechanistic explanation (116). Most cases of ICANS can be managed successfully with a short course of corticosteroids. There has been some concern that the steroids may affect CAR T cell function and quality, but thus far steroid use to control ICANS does not appear to affect clinical outcomes (117). Due to CRS and ICANS associated with CAR T and bsAbs, their administration in MM has largely been restricted to transplant centers experienced in managing these syndromes.

## Discussion

The advent of the first MoAb with clinical efficacy in myeloma, daratumumab, led to an explosion of new research on methods to harness the host’s immune system to enhance treatment response and overall survival (118). BsAbs and CAR T have given new options and hope for patients running out of treatment choices after failing PI, IMiD, and anti-CD38 antibody therapies. However, each therapeutic modality has unique pros and cons which could be more or less suitable for different patient populations. Bispecific antibodies offer the advantage of being “off the shelf” products that can be used promptly to treat MM, whereas CAR T administration involves a 4-6 week process of lymphocyte collection, manufacturing, and infusion (119). During these 4-6 weeks “bridging” chemotherapy or immunotherapy may need to be used to prevent disease progression while patients are waiting (120). While CAR T production takes time, it is typically a single infusion with a substantial depth and duration of response, even in the most refractory of patients (93, 110). On the other hand, bsAbs require regular dosing to maintain efficacy, meaning they are eventually less convenient to patients through ongoing treatment in clinic (121).

There remain many outstanding clinical questions about the role of T cell-mediated treatment in MM. The optimal timing sequence for CAR T and bsAbs is unknown. Potential roles for T cell therapies could include consolidation after induction chemotherapy or ASCT in an effort to achieve the deepest possible remissions or, alternatively, as salvage treatment in those patients failing IMiDs and PIs. Trials of CAR T and bsAbs in early phases of myeloma treatment of MM are also currently ongoing. It is also unknown how much the use of a prior anti-BCMA targeted therapy such as anti-BCMA CAR T affects the efficacy of a subsequent anti-BMCA treatment with a different modality such as bsAbs, and vice-versa. CAR T therapy at this point does not appear to be curative, and relapses may occur due to selection of BCMA-negative MM cells as well as antigen escape *via* secretion of BCMA into the bloodstream through the action of gamma-secretase (88). Gamma-secretase inhibitors are currently being tested with anti-BCMA CAR T to combat this potential mechanism of resistance (122). Already, we have seen an increase in activity when the antibody-drug conjugate belantamab is combined with the PI bortezomib. More than likely, combination therapies of IMiDs, PIs, bsAbs, and CAR T will be used in the future. Treatments will be tailored to be patient and tumor-type specific. Newer technologies involving trivalent CAR T, CAR-NK cells, and more advanced co-stimulatory domains are under exploration and may enhance the efficacy and reduce the toxicity of immunotherapy. Truly, it is an exciting era in MM therapy with a brighter future for patients.

## Author Contributions

AK and TM wrote the manuscript. PF and DS aided in revisions. All authors read and approved the submitted version.

## Funding

DS is supported by a grant from the National Cancer Institute (K08CA222704).

## Conflict of Interest

The authors declare that the research was conducted in the absence of any commercial or financial relationships that could be construed as a potential conflict of interest.

## Publisher’s Note

All claims expressed in this article are solely those of the authors and do not necessarily represent those of their affiliated organizations, or those of the publisher, the editors and the reviewers. Any product that may be evaluated in this article, or claim that may be made by its manufacturer, is not guaranteed or endorsed by the publisher.
